# Assessment of managerial processes applied in multinational special education institutions

**DOI:** 10.1016/j.heliyon.2023.e19514

**Published:** 2023-08-30

**Authors:** Kazım Küçükalkan, Mukaddes Sakallı Demirok, Mustafa Avcin

**Affiliations:** a Department of Special Education, Near East University, Nicosia, Cyprus; b Business Administration Department, Rauf Denktas University, Nicosia, Cyprus

**Keywords:** Educational management, Managerial processes, Special education, Special education institution, Special education teacher

## Abstract

The aim of this research is to evaluate and analyze the managerial processes applied in special education institutions operated by public and private enterprises that provide special educational needs in different countries to determine problems and develop solution proposals with a new educational management model. The quantitative data for the study were collected using the "Management Processes Scale in School Management” which was created by Gregg, and the data used in the research belong to the academic years 2020–2021. In line with the collected data, the views of 45 managers and 130 teachers across North Cyprus, Turkey, and the UK were collated. Empirical results imply that all managerial processes are strongly related to the teachers' perspective, and for managers' decision-making, organization, evaluation are favorably related to effective management in all countries.

## 1 Introduction

Individuals with special needs can only benefit from special education given at the highest level, depending on the effective management of special education institutions [1]. However, there are various problems in the managerial processes of special education institutions [2,3,4,5]. Boscardin [6], Crocket [7], Thornton, Peltier and Medina found that the realization of educational objectives expected from special education institutions can only be achieved through effective management, qualified managers, and teachers [8]. Thus, this will ensure that institutions providing special education services are managed in accordance with the expected objectives, that individuals with disabilities or giftedness will benefit from more educational opportunities, and that inequalities towards these individuals will be minimized [6,7,8].

Conducted research with managers of special education institutions serving abroad; it was observed that there are various managerial problems and the competencies of those managers who carry out management activities are questioned [6]. It is stated that school managers with no experience are appointed to special education schools and do not have the leadership skills to manage schools or field experience in special education [9]. Moreover, instructional leadership preparation programs run very slowly [7]; the cooperation between teachers and managers is not sufficient [10]; the use of different tools and equipments in special education services entails an obligation to employ different personnel the need to take special precautions in physical structures increases the cost element for these institutions, alongside allocating special resources [11]. In order to solve the problems related to management, the managerial processes should be looked at holistically, the relations between the problems should be evaluated and solutions should be produced [11].

Along with the existence of many different classifications found in literature, the research of Başaran [12], Özdemir [13] and Şanlı [14] have also shown that Gregg's education system is affected by decision-making, planning, organization, communication, influencing, coordination, and evaluation, which is based on the "Managerial Processes Classification in School Management" that he has gained. In this case, necessary data on managerial processes implemented by managers of special education institutions working in different countries were gathered with the participation of institution managers and special education teachers. Although many studies exist focusing on the problems experienced in the field of special education, no study has been found that evaluates the managerial processes applied in special education institutions in different countries, including the subject of comparative education related to the managerial processes of special education institutions.

This study will contribute to the literature as it is based on the assessment of the managerial processes applied in special education institutions and focuses on the problems experienced in the field of special education. Therefore, the study evaluates the problems regarding managerial processes applied in multinational special education institutions, and analyses the problems experienced, culminating in the creation of solution proposals for those problems.

### 1.1 Special education and special education institutions

Special education is the education given by specially trained personnel in specially designed environments to individuals who show different developmental patterns compared to their normally developing peers [5]. The expression "individuals with special needs" is an integrative concept since it includes individuals with behavioral, cognitive, sensory, learning, and social problems as well as gifted individuals [15,16]. Therefore, individuals have unique emotional characteristics, different physical structures, functionality, different learning speeds, different learning characteristics, and students with these differences can benefit from general education services within certain limits and the size of the differences increases, the education provided is insufficient, and the necessity of organizing special education services comes to the fore [17]. The principle of equal opportunity in education is one of the factors that make up the basic building blocks of democratic and contemporary societies [18]. According to Akçamete, Büyükkarakaya, Bayraklı and Yıldırım, the purpose of providing special education is to allow each individual with special needs to become social and self-sufficient individuals who can use their capacities at the highest level, in line with their educational needs, competencies, interests, abilities, and a social life [19].

Special education institutions are defined as educational institutions operated by public institutions or private enterprises to provide educational support to individuals in need of special education, to prepare them for life, to enable them to acquire a job and profession, or to meet the learning needs of those who are unable to benefit from formal education programs by developing basic life skills [20,5]. In addition, it allows individuals with special needs who are suitable for education together with their normally developing peers to continue their education and training through inclusive education [21]. Among the important factors affecting the quality of the education provided in all institutions providing special education are the physical characteristics, competencies of the institutions, opportunities in the physical environment inside and outside the institution, and managerial processes [[22], [23]].

### 1.2 Educational management

Although education provides permanent learning, it is an important lifelong process in terms of reaching modern society's standards and ensuring the sustainability of quality of life [24]. Cozoğlu states that, one of the main purposes of education is to create learning educational institutions based on a systematic and planned process, that the managers in these institutions see themselves as a part of the institution, attach importance to the learning, opinions and needs of personal and corporate employees, and have high communication skills and states that there are leaders who support the permanent development of the institution [25]. The process of applying educational management to a more limited area, namely the school, constitutes school management. The manager is in responsibility of personnel management, security procedures, accounting, finance, and other services at the school [26,27]. The excellent capabilities of the school management are critical to the school's performance [[14], [28]].

The aim of educational management is to implement education policies determined by the state by using human and material resources in the most effective way in order to achieve the determined goals of educational institutions, and institution managers are responsible for managing their institutions within the framework of the general objectives and basic principles of national education and the specific objectives of the institutions [29,25,30]. Russel T. Gregg made the most used managerial processes classification in 1957 as; decision-making, planning, organization, communication, influencing, coordination and evaluation [31]. The effective implementation of managerial processes ensures the success of schools [32].

### 1.3 Managerial processes in special education institutions

#### 1.3.1 Decision-making process

Decision-making entails identifying potential solutions to problems, avoiding or resolving conflicts, interpreting information about a situation or problem, and reaching a conclusion [4,31]. A good decision is one that will achieve the goals of the organization or institution in a scientific, clear, and precise way that can be easily understood by those who apply it and comply with the legislation [33]. While making a decision, it is necessary to understand exactly what the problem is, collect information about the problem, analyze the information, then interpret it, evaluate the options for the solution, find the best solution among the options, apply it, and finally evaluate [34]. School managers will have to make decisions about many subjects during school management, and any decision they make will ultimately have an influence on teachers, students, employees, and the performance of the school [33]. Some reasons, such as the lack of information from the decision-maker, limited time, and a lack of a healthy communication system on the issues to be decided, can make it difficult to take realistic decisions [34].

#### 1.3.2 Planning process

As in every organization, the function of management applied in schools should be operated in a planned manner in order to ensure the effectiveness and sustainability of the school [35]. The steps in the planning process include: "1-team building and consensus; 2-assessment of the resources available; 3-analysis of the economic, political, and social environments; 4-forecasting and evaluation of the influence impression of possible future developments; 5-deciding what the objectives will be by creating a common vision; and 6-deciding on the practices that need to be done in order to achieve the objectives" [36]. Planning forces school managers to look ahead, enables managers to predict change and give more appropriate responses, and reduces uncertainty [36]. Well-done planning will prevent problems and crises for managers and will also facilitate the achievement of the goals of the manager and the institution [37].

#### 1.3.3 Organization process

Eren described the organizing is the process of combining the human and other resources of the organization in order to achieve organizational goals and direct these resources [38]. Organization process is determining what the necessary tasks are in order to achieve the objectives and then determining the people who will fulfill these tasks, taking into account the nature of the activities [38]. Many factors are taken into account in order for educational institutions to have an effective organizational culture, such as the communication of all employees in the school with each other, how they develop themselves, their expectations of students, student behaviors, and communication of parents with school employees [39].

#### 1.3.4 Communication process

Communication, which is one of the most important elements for the future of the institution, is the process of influencing information, feelings, thoughts, meanings, attitudes, and behaviors between two or more people [40,35]. According to Hoy and Miskel, it is important to establish a healthy communication process between managers, teachers, employees, students, and the external environment in order for the institution to achieve its goals, motivate employees and students, make the right decisions, ensure coordination, and carry out healthy audit activities [[4], [41]]. On the other hand, plans made and the right decisions taken to achieve goals will be possible by establishing a healthy communication process [42].

#### 1.3.5 Coordination process

According to Bursalıoğlu, as organizations develop, the number of employees increases, different statuses are formed, coordination between individuals and between statuses becomes difficult, and the importance of coordination processes for the organization to achieve its goals becomes apparent [34]. Effective and successful managers fulfill the duties of creating an appropriate organizational climate, selecting and training talented subordinates, preparing integrated plans and programs that their subordinates can implement, and implementing an effective management system [20]. The main task of the education manager is to manage the coordination process effectively by combining the human and material resources separated from each other by the division of labor, reconciling knowledge and skills, and directing the educational institutions to realize their organizational, managerial, and educational goals [29,35].

#### 1.3.6 Influence process

A manager who wants to manage the influencing process in the best way " … should have the necessary information about all the employees they are responsible for; constantly monitor the social structure within the organization; not lose their broad perspective by obsessing over the details; and in order to ensure rights and justice between the organization and the employees" [43]. According to Sertkaya, managers are making an effort to provide effective guidance, and it is necessary to pay attention these principles: "1. Employees should be placed in the most suitable positions for their knowledge, skills, and abilities. 2. Managers should convey their experiences to their employees, and set a good example for them. 3. Team spirit should be created. 4. When necessary, participation in the decisions to be taken should be practiced. 5. Employee relations with the institution should be monitored. 6. There should be internal communication networks. 7. There should be an audit." [44].

#### 1.3.7 Evaluation process

The evaluation process, which is the final step in the managerial process, is the process of reviewing the results obtained after organizational decisions, planning, communication, and coordination in order to determine the level of realization of organizational goals and the extent of consistency [4,35]. Managers should set aside prejudices in order to be able to make an impartial and valid evaluation, be clear when setting goals, choose the most appropriate education and training methods in order to achieve the goals, and also have the necessary skills and equipment in order to fulfill the evaluation task [[32], [45]].

## 2 Materials and methods

### 2.1 Research pattern and model

**Fig. 1:**
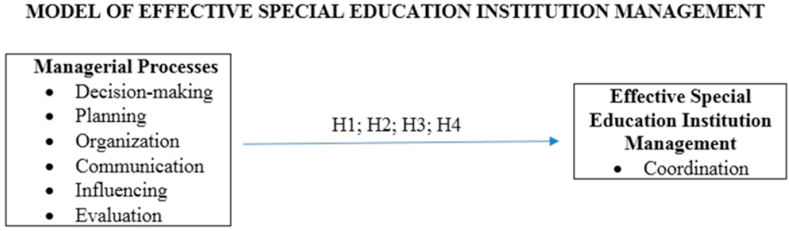
Source:Author’s own.

In this research, a quantitative research method and a relational screening model were used. Relational screening models are aiming to determine the presence and/or level of change between two or more variables and to specify the relationships between the variables [46]. The variables included in the model are as follows; “Independent variables are decision-making, planning, organization, communication, influencing, evaluation and dependent variable is coordination. The model variables are presented in Fig. 1 and explain in the findings section.

The method of quantitative research was implemented for the study with a focus on the correlational research design. The aim of using the correlational research approach was to investigate the degree to which variations in one or more variables correspond with other variations in one or more factors based on correlation coefficients. It is concerned with discovering or clarifying relationships among variables using the correlation coefficient. The design of correlational research was adopted since the research was planned to examine the strength and nature of the relationship that exists between managerial processes and the effective management of special education institutions in different countries. By using the data obtained from teachers/managers and collecting/correlating the analysis of this data, we are able to determine our new educational management model in special education institutions.

The hypotheses of the research are as follows: “1-Hypothesis 1. There is a positive relationship between the managerial processes against effective special education institution management, according to special education teachers’ opinions and demographic structures; Hypothesis 2. There is a positive relationship between the managerial processes against effective special education institution management, according to special education institution managers’ opinions and demographic structures; Hypothesis 3. Managerial processes of decision-making, communication, organization, evaluation, planning, and influencing have an impact on effective special education institution management when teachers are concerned in North Cyprus, Turkey, and the UK; Hypothesis 4. Managerial processes of decision-making, organization, and evaluation have more impact on effective special education institution management when managers are concerned in North Cyprus, Turkey, and the UK.”

### 2.2 Sampling and data collection

#### 2.2.1 Sampling

The universe of the research consists of special education teachers and institution managers working in special education institutions operated by public and private enterprises in North Cyprus, Turkey, and the UK in the 2020–2021 educational year. The research consisted of 89 special education teachers and 12 institution managers from North Cyprus; 102 special education teachers and 56 managers from Turkey; 46 special education teachers and 9 managers from the UK. In total, 237 special education teachers and 77 institution managers participated. In addition, participants in the research were rearranged with the criterion sampling method, which is one of the non-probabilistic, purposeful sampling methods, formed by the individuals determined in line with the aims of the research. Using this method, missing data imputations and branch teachers were excluded from the research. As a result, 50 special education teachers and 10 managers from North Cyprus, 60 special education teachers and 30 managers from Turkey, and 20 special education teachers and 5 managers from the UK formed the sample of the research. The measurement data of the scale include 130 special education teacher and 45 institution managers in total. Although the sample size of the study was small, the confidence interval in this study was determined to be 95%. The demographic characteristics of the participants are presented in Table 1.

**Table 1:** Demographic characteristics of the participants.

Institution Managers		North Cyprus						Turkey						UK						
		Special Education Institution (Public)		Special Education Institution (PE^a^)		Total		Special Education Institution (Public)		Special Education Institution (PE^a^)		Total		Special Education Institution (Public)			Special Education Institution (PE^a^)			Total
		f	%	f	%	f	%	F	%	f	%	f	%	f	%	f		%	f	%
Gender	Female	4	57,1	0	0	4	40	10	55,6	8	66,7	18	60	2	100	3		100	5	100
	Male	3	42,9	3	100	6	60	8	44,4	4	33,3	12	40	0	0	0		0	0	0
	Total	7	100	3	100	10	100	18	100	12	100	30	100	2	100	3		100	5	100
Age	20–25	0	0	1	33,3	1	10	3	16,6	0	0	3	10	0	0	0		0	0	0
	26–30	2	28,7	0	0	2	20	5	27,8	3	25	8	26,7	0	0	0		0	0	0
	31–35	3	42,6	1	33,3	4	40	5	27,8	0	0	5	16,7	0	0	0		0	0	0
	36–40	0	0	0	0	0	0	1	5,6	3	25	4	13,3	0	0	0		0	0	0
	41 and upper	2	28,7	1	33,3	3	30	4	22,2	6	50	10	33,3	2	100	3		100	5	100
	Total	7	100	3	100	10	100	18	100	12	100	30	100	2	100	3		100	5	100
Education Level	Associate Degree	0	0	0	0	0	0	4	22,2	2	16,7	6	20	0	0	0		0	0	0
	Bachelor Degree	1	14,3	2	66,7	3	30	9	50	7	58,3	16	53,3	2	100	3		100	5	100
	Master Degree	3	42,9	1	33,3	4	40	5	27,8	3	25	8	26,7	0	0	0		0	0	0
	PHD	3	42,9	0	0	3	30	0	0	0	0	0	0	0	0	0		0	0	0
	Other	0	0	0	0	0	0	0	0	0	0	0	0	0	0	0		0	0	0
	Total	7	100	3	100	10	100	18	100	12	100	30	100	2	100	3		100	5	100
Teaching Experience	0–5 year	1	14,3	1	33,3	2	20	8	44,4	2	16,7	10	33,3	0	0	1		33,3	1	20
	6–10 year	0	0	0	0	0	0	6	33,3	4	33,3	10	33,3	0	0	1		33,3	1	20
	11–15 year	3	42,7	1	33,3	4	40	2	11,1	3	25	5	16,7	1	50	0		0	1	20
	16–20 year	1	14,3	1	33,3	2	20	2	11,1	0	0	2	7	0	0	0		0	0	0
	21 year and upper	2	28,7	0	0	2	20	0	0	3	25	3	10	1	50	1		33,3	2	40
	Total	7	100	3	100	10	100	18	100	12	100	30	100	2	100	3		100	5	100
Job Role	Manager	1	14,3	3	100	4	40	3	16,7	6	50	9	30	2	100	3		100	5	100
	Assistant Manager	0	0	0	0	0	0	4	22,2	1	8,3	5	16,7	0	0	0		0	0	0
	Responsible Teacher	6	85,7	0	0	6	60	11	61,1	5	41,7	16	53,3	0	0	0		0	0	0
	Total	7	100	3	100	10	100	18	100	12	100	30	100	2	100	3		100	5	100
Special Education Teachers		North Cyprus						Turkey						UK						
		Special Education Institution (Public)		Special Education Institution (PE^a^)		Total		Special Education Institution (Public)		Special Education Institution (PE^a^)		Total		Special Education Institution (Public)			Special Education Institution (PE^a^)			Total
		f	%	f	%	f	%	f	%	f	%	f	%	f	%	F		%	f	%
Gender	Female	33	82,5	7	70	40	80	31	86,1	19	79,2	50	83	5	71,4	12		92,3	17	85
	Male	7	17,5	3	30	10	20	5	13,9	5	20,8	10	17	2	28,6	1		7,7	3	15
	Total	40	100	10	100	50	100	36	100	24	100	60	100	7	100	13		100	20	100
Age	20–25	5	12,5	4	40	9	18	5	13, 9	7	29,2	12	20	1	14,3	1		7,7	2	10
	26–30	20	50	3	30	23	46	5	13, 9	2	8,3	7	12	3	42,9	1		7,7	4	20
	31–35	12	30	2	20	14	28	9	25	3	12,5	12	20	0	0	1		7,7	1	5
	36–40	1	2,5	1	10	2	4	6	16,8	7	29,2	13	21	1	14,3	0		0	1	5
	41 and upper	2	5	0	0	2	4	11	30,7	5	20,8	16	27	2	28,6	10		76,9	12	60
	Total	40	100	10	100	50	100	36	100	24	100	60	100	7	100	13		100	20	100
Education Level	Associate Degree	0	0	0	0	0	0	7	19,4	3	12,5	10	17	0	0	1		7,7	1	5
	Bachelor Degree	31	77,5	8	80	39	78	22	61,1	19	79,2	41	68	4	57,1	6		46,2	10	50
	Master Degree	9	22,5	1	10	10	20	6	16,7	2	8,3	8	13	2	28,6	3		23,1	5	25
	PHD	0	0	1	10	1	2	1	2,8	0	0	1	1,7	0	0	0		0	0	0
	Other	0	0	0	0	0	0	0	0	0	0	0	0	1	14,3	3		23,1	4	20
	Total	40	100	10	100	50	100	36	100	24	100	60	100	7	100	13		100	20	100
Teaching Experience	0–5 year	24	60	5	50	29	58	16	44,4	11	45,8	27	45	2	28,6	4		30,8	6	30
	6–10 year	9	22,5	4	40	13	26	9	25	4	16,7	13	22	3	42,9	1		7,7	4	20
	11–15 year	5	12,5	0	0	5	10	4	11,1	2	8,3	6	10	1	14,3	3		23,1	4	20
	16–20 year	1	2,5	1	10	2	4	4	11,1	4	16,7	8	13	1	14,3	1		7,7	2	10
	21 year and upper	1	2,5	0	0	1	2	3	8,3	3	12,5	6	10	0	0	4		30,8	4	20
	Total	40	100	10	100	50	100	36	100	24	100	60	100	7	100	13		100	20	100
Job Role	Manager	33	82,5	7	70	40	80	31	86,1	19	79,2	50	83	5	71,4	12		92,3	17	85
	Assistant Manager	7	17,5	3	30	10	20	5	13,9	5	20,8	10	17	2	28,6	1		7,7	3	15
	Responsible Teacher	40	100	10	100	50	100	36	100	24	100	60	100	7	100	13		100	20	100
	Total	5	12,5	4	40	9	18	5	13, 9	7	29,2	12	20	1	14,3	1		7,7	2	10

a Special education institutions operated by private enterprises (PE).

#### 2.2.2 Data collection

The scale used in the collection of data was first created by Gregg [47], "Management Processes Scale in School Management" and later developed and updated by Ebabil in her research titled "Evaluation of the Progress of the Management Processes in Pre-School Education Institutions Based on the Views of Managers and Teachers" [37]. The scale consists of 63 questions, and two questions were removed in line with expert opinions due to not being suitable for the aims of the research. Each question in the scale was subjected to a 5-point Likert-type rating, and the levels of participation in the questions in the scale were "Never = 1," "Very Rarely = 2," "Occasionally = 3," "Often = 4", and "Always = 5.". In order to balance the negative consequences of the COVID-19 pandemic on the process, the scale used in the research was arranged using "Google Forms" and data was collected by providing access to this platform without face-to-face interviews with the participants. According to Baytak, online surveys provide many advantages, such as quick access and analysis, that provide the opportunity to reach a large number of participants in a short space of time [48]. Then, the collected data is transferred to computer software in an orderly manner. The data that was decided not to be included in the research in accordance with the criterion sample was removed, and numerical and statistical analysis were conducted using the SPSS-26 program with the included data.

### 2.3 Analysis

The steps of content formation are as follows: In the research, firstly, frequency and percentage values were calculated for the distribution of the participants according to their demographic characteristics. Measures such as mean and variance are derived from raw data in parametric testing, and procedures are carried out. Raw data is sorted and sequence numbers are assigned in non-parametric testing. These sequence numbers are used for transactions. Non-parametric tests are less effective than parametric testing. Therefore, t-tests and one-factor analysis of variance (ANOVA) were conducted to compare the opinions of managers and special education teachers working in special education institutions in different countries regarding the sub-dimensions of the scale (decision-making, planning, organization, communication, influencing, coordination, and evaluation). Later, the average of the questions related to each dimension was used in the formation of sub-dimension scores. In addition, the average scores of the survey were calculated, and the *t*-test for the variables containing two groups and the ANOVA for the variables containing more than two groups were applied to compare the opinions of managers and special education teachers. As a result of the analysis of variance, if there is a difference between the groups, Tukey, one of the multiple comparison tests, and the Scheffe test, which is the most frequently used since the number of groups is different, were used in the data collected by the scale in social sciences to find the source of the difference. Data was analyzed using descriptive statistics such as mean and standard deviation, and inferential statistics such as Pearson Product Moment Correlation and Multiple Linear Regression analyses where applicable. The results of the final stages of data analysis are also interpreted. In the detailed section of the findings, the data is processed to the finest detail. The obtained data is reviewed and interpreted according to the quantitative research method. Based on these results, proposals were made by researchers who will conduct research on the subject, and the results are supported by relevant literature.

### 2.4 Model specification

The models of this study are specified based on the general multiple linear regression equation given: Y’ = a + b1X1 + b2X2 +b3X3 … bnXn. …. …. …. …. …. …. …. …. . i

Where

a = The intercept (the value of Y when all predictor variables are equal to 0)

Y’ = the predicted value of the dependent variable

b1 – bn = Unstandardized regression weights

X1 – Xn = Predictor variables

### 2.5 Findings

In this research, 3 different situations covering special education institutions' managerial processes in different countries were discussed and presented in the findings. Independent sample *t*-test and ANOVA tests were used to determine whether there was a significant difference between the means in the study. The themes covered in the research are presented as follows:

#### 2.5.1 According to special education teachers’ opinions and demographic variables of teachers working in special education institutions operated by public and private enterprises, what is the relationship between the managerial processes against effective special education institution management?

According to special education teachers’ opinions, the managerial processes about teaching experience variable, there is a positive relationship between managerial processes and effective special education institution management. The coordination (1,265), evaluation (0,982), and influencing (0,783) processes in North Cyprus, evaluation (1,303), communication (1,064), and coordination (0,895), processes in Turkey, communication (0,584), evaluation (0,531) and planning (0,442) managerial processes for the UK are strongly significant, and had a great impact on effective management in special education institutions.

About the education level variable, coordination (1,276), communication (1,188), and evaluation (0,956) processes in North Cyprus, evaluation (1,222), influencing (1,104), and communication (0,740) processes in Turkey and for the UK, evaluation (2,309), communication (2,239), and coordination (1,944) managerial processes are strongly significant and have a great impact on effective management in special education institutions.

With the managerial processes for the age variable, there is a positive relationship between managerial processes and effective special education institution management. The results show that, planning (1,731), coordination (1,337), and evaluation (1,303) processes in North Cyprus, planning (1,640), communication (1,547), and evaluation (1,454) in Turkey and for the UK, communication (1,663), coordination (1,333), and evaluation (1,200) managerial processes are strongly significant and have a great impact on managerial processes against institutional management.

The outcomes of managerial processes for school types implemented by private enterprises special education schools (PE) and public special education institutions. According to the results, organization (4,333), communication (4,238), and planning (4,231) processes strongly determine effective institutional management in public special education institutions and evaluation (4,000), organization (3,950), and coordination (3,914) processes are strongly associated with effective special education institution management in PE in North Cyprus. In Turkey, organization (4,180), coordination (4,053), communication (4,028) processes in public and communication (4,435), organization (4,368), and planning (4,288) managerial processes in PE are strongly associated with effective special education institution management. Finally, communication (3,200), evaluation (3,185), and organization (2,813) processes in public and evaluation (3,492), communication (3,486), and coordination (3,296) processes are strongly associated with effective special education institution management in PE in the UK.

On the other hand, organization (4,231), communication (4,140), and planning (4,133) processes strongly determine effective special education institution management by females and organization (4,550), communication (4,450), and coordination (4,400) by males in North Cyprus. In Turkey, organization (4,235), communication (4,116), and coordination (4,088) processes by female special education teachers and communication (4,264), organization (4,227), and coordination (4,130) managerial processes by males strongly determine effective special education institution management. Finally, in the UK, evaluation (3,294), communication (3,271), and coordination (3,025) managerial processes strongly determine effective special education institution management by female special education teachers and communication (4,133), evaluation (4,000), and coordination (3,762) by males.

#### 2.5.2 According to special education institution managers’ opinions and demographic variables of the managers working in special education institutions operated by public and private enterprises, what is the relationship between the managerial processes applied in special education institutions against effective special education institution management?

According to special education institution managers’ opinions, the managerial processes for the teaching experience variable, there is a positive relationship between managerial processes and effective special education institution management. Influencing (0,356), evaluation (0,227), and communication (0,158) in North Cyprus, planning (0,803), influencing (0,518), and decision-making (0,380) in Turkey and for the UK, planning (0,469), influencing (0,359), and coordination (0,210) processes are strongly significant and have a great impact on effective management in special education institutions.

And, influencing (0,902), coordination (0,315), and communication (0,311) in North Cyprus, coordination (0,618), planning (0,465), evaluation (0,461) in Turkey, and finally, in the UK, planning (0,469), influencing (0,359), and coordination (0,210) managerial processes are strongly significant against institutional management and have a great impact on managerial processes that determine effective management in special education institutions.

According to the age variable results, in North Cyprus, influencing (1,809), evaluation (1,593), coordination (0,893), and in Turkey, planning (1,736), coordination (0,584), and evaluation (0,501) managerial processes are strongly significant against institutional management. In the UK, the participants of the study were in a single age group (41 and over), and at the same time, the number of variables was single. Because of that, the analysis was not performed.

On the other hand, in public special education institutions, communication (4,529), organization (4,464), and planning (4,414) processes in North Cyprus, coordination (4,667), organization (4,569), planning (4,328) processes in Turkey, organization (4,563), evaluation (4,500), planning (4,450) processes in the UK and in special education institutions operated by private enterprises (PE), evaluation (4,852), communication (4,700), coordination (4,619) processes in North Cyprus, communication (4,392), organization (4,240), evaluation (4,046) processes in Turkey, communication (4,533), evaluation (4,519), organization (4,333) managerial processes in the UK are strongly associated with effective institution management.

By females, communication (4,483), planning (4,433), coordination (4,405), and by males, evaluation (4,833), communication (4,725), coordination (4,643) processes in North Cyprus, by females, organization (4,389), coordination (4,357), communication (4,311) processes, and by males, organization (4,510), coordination (4,452), communication (4,300) processes in Turkey and finally, in the UK, evaluation (4,511), influencing (3,486), and communication (4,480) processes strongly determine effective special education institution management by female managers. There weren't any male special institution manager participants in the UK for this survey.

The psychometric qualities of a test are linked to the assessment data, which determines how effectively it measures the parameters' interest. As a result, numerical measures such as a coefficient or an index are frequently used to describe the tests. Furthermore, the construction of a good psychometric test is dependent on the amount to which it is statistically analyzed, which guarantees that it has the necessary psychometric features. The investigation carried out first with factor analysis which determined the formulation of the model to test the hypothesis using regression analysis.

Factor analysis has been carried out to determine the independent variables against the dependent variable. The results of the factor analysis regarding the survey questions for special education teachers are displayed in Table 2, Table 3, Table 4 and for institution managers are shown in Table 6, Table 7, Table 8.

**Table 2:** Factor analysis results of north Cyprus.

Managerial Processes	North Cyprus			
	Question Statement	Factor	%	Reliability
Decision-making	Makes decisions after meeting with both parents and staff to solve institutional problems. (1)	0,771	19,514	0,918
	Consults with staff in order to obtain beneficial sources of information from relevant publications and people. (4)	0,766		
	Makes decisions together with staff for determining, regulating and supporting productive usage of equipment and fixtures in line with the needs of the institution. (5)	0,848		
	During the decision-making process he behaves in a democratic and participatory manner. (6)	0,871		
Planning	Ensures that all the precautions relating to the cleanliness and order of the institution are taken. (11)	0,779	41,901	0,863
	Plans schedules regarding families (parent meeting dates, family education seminars etc.) at the teacher’s council meeting. (20)	0,779		
Organization	Explains duties, roles and responsibilities of the institution to staff (teachers and other employees) in a written and clear way. (21)	0,875		0,965
	Ensures that all institutional related documents are processed in time and announced to institutional employees. (23)	0,922		
	There is a division of labor between teachers and other staff. (24)	0,905		
	Organizes the institution appropriately, in consideration and parallel to the institutions goals and objectives. (25)	0,948		
	By building good relations with the environment he benefits from both people and groups in parallel with an institution aims. (27)	0,887		
Coordination	Encourage cooperation between teachers, other employees, students, parents, school council and top-level managers to assist in the institution reaching its goals. (40)	0,772		0,845
	Schedules educational activities for teachers (monthly-daily plans, parent meetings, family education and celebrating special days) and ensure that all the activities are carried out collaboratively. (41)	0,749		
	Prioritize in-service education and ensure that all the staff members are trained well in this field. (42)	0,766		
Communication	Benefits from clear and effective communication methods and tools while communicating with teachers. (29)	0,634	24,276	0,776
	Ensures that a warm and friendly atmosphere is created between staff relations within the institution. (34)	0,634		
Evaluation	Monitors and evaluates teacher’s lectures and work in terms of their efficiency at various times of the academic year. (53)	0,588		0,900
	Evaluates teachers objectively and equally. (57)	0,872		
	Displays constructive and developing behavior when delivering results of an audit and its evaluation. (58)	0,905		
	Sees the institution as a whole and assess and evaluate the effectiveness and efficiency level of the school. (59)	0,817		

*Note: The numbers in the table indicate the order of the question statements in the questionnaire*.

**Table 3:** Factor analysis results of Turkey.

Managerial Processes	Turkey			
	Question Statement	Factor	%	Reliability
Decision-making	Makes decisions together with staff for determining, regulating and supporting productive usage of equipment and fixtures in line with the needs of the institution. (5)	0,832	20,568	0,879
	During the decision-making process he behaves in a democratic and participatory manner. (6)	0,768		
	Ensures all staff attend meetings in relation to decisions taken about the institution. (9)	0,710		
Planning	Takes into consideration staff opinions while planning cultural activities and sightseeing trips. (13)	0,776		0,874
	Takes into consideration environmental expectations and opportunities while planning educational activities. (16)	0,776		
Organization	Explains duties, roles and responsibilities of the institution to staff (teachers and other employees) in a written and clear way. (21)	0,921	64,785	0,980
	Ensures that all institutional related documents are processed in time and announced to institutional employees. (23)	0,959		
	There is a division of labor between teachers and other staff. (24)	0,933		
	Organizes the institution appropriately, in consideration and parallel to the institutions goals and objectives. (25)	0,950		
	Plans the institutions activities in compliance with effective usage of both human and pecuniary resources. (26)	0,943		
Coordination	Ensures that all the institutions members participate and play an active and productive role in the education mechanism of the institution. (39)	0,976		0,978
	Schedules educational activities for teachers (monthly-daily plans, parent meetings, family education and celebrating special days) and ensure that all the activities are carried out collaboratively. (41)	0,959		
	Informs institution employees regarding each other’s work. (44)	0,925		
Communication	Benefits from clear and effective communication methods and tools while communicating with teachers. (29)	0,861		0,978
	Does works for the development of communication between staff. (30)	0,931		
	Promptly notifies teachers of any change in the legislations and application. (31)	0,912		
	Informs teachers regarding newly received directions and procedures. (32)	0,920		
	Uses vertical and horizontal communication types in the institution. (33)	0,926		
	Follows the course of any activities organized in the institution and consult with teachers about any observed deficiencies and measures to be taken. (35)	0,909		
	Allow teachers to express their ideas and opinions clearly within the institution. (36)	0,921		
	Benefits from clear and effective communication methods and tools while communicating with teachers. (38)	0,911		
Influencing	Assists teachers and other employees about embracing their work and fully committing themselves to the institution. (49)	0,917		0,957
	Motivates teachers for using various education methods and techniques. (51)	0,917		

Note: The numbers in the table indicate the order of the question statements in the questionnaire.

**Table 4:** Factor analysis results of UK.

Managerial Processes	UK			
	Question Statement	Factor	%	Reliability
Communication	Does works for the development of communication between staff. (30)	0,975	100	0,989
	Uses vertical and horizontal communication types in the institution. (33)	0,975		
	Allow teachers to express their ideas and opinions clearly within the institution. (36)	0,960		
	Ensures that the wishes and complaints of staff in charge of teachers are easily conveyed to the institutions management. (37)	0,986		
Influencing	Uses reward as a motivation source to boost/increase teachers’ success. (46)	0,932		0,952
	Uses menace and punishment to motivate teachers. (47)	0,899		
	Finds immediate and effective solutions to the problems experienced within the institution. (50)	0,871		

Note: The numbers in the table indicate the order of the question statements in the questionnaire.

**Table 5:** Measure of factor analysis applicability of north Cyprus, Turkey, and the UK.

			North Cyprus	Turkey	UK
Measure of Factor Analysis Applicability	Kaiser-Meyer-Olkin Measure of Sampling Adequacy		0,850	0,736	0,908
	Bartlett Test of Sphericity	Chi-Square Df Significance	1473,834	3134,211	236,72
			190	253	21
			0,000	0,000	0,000

**Table 6:** Factor analysis results of north Cyprus.

Managerial Processes	North Cyprus			
	Question Statement	Factor	%	Reliability
Coordination	I prioritize in-service education and ensure that all the staff members are trained well in this field. (42)	0,949	100	0,922
	I play a conciliatory role between teachers, top-level managers (ex. director of national education) students, parents and institution staff. (43)	0,949		
Evaluation	I monitor and evaluate teacher’s lectures and work in terms of their efficiency at various times of the academic year. (53)	0,913		0,930
	I implement the results of inspections and evaluations together with teachers. (55)	0,913		

Note: The numbers in the table indicate the order of the question statements in the questionnaire.

**Table 7:** Factor analysis results of Turkey.

Managerial Processes	Turkey			
	Question Statement	Factor	%	Reliability
Decision-making	I make decisions about education and training (monthly, daily plan, parents meeting, family education etc.) together with teachers. (3)	0,786	100	0,919
	I consult with staff in order to obtain beneficial sources of information from relevant publications and people. (4)	0,907		
	I make decisions together with staff for determining, regulating and supporting productive usage of equipment and fixtures in line with the needs of the institution. (5)	0,869		
	During the decision-making process I behave in a democratic and participatory manner. (6)	0,759		
Organization	I plan the institutions activities in compliance with effective usage of both human and pecuniary resources. (26)	0,867		0,912
	I frequently gather teacher’s council to find solutions regarding teaching and education in parallel with institution objectives. (28)	0,867		

Note: The numbers in the table indicate the order of the question statements in the questionnaire.

**Table 8:** Factor analysis results of UK.

Managerial Processes	UK			
	Question Statement	Factor	%	Reliability
Coordination	I ensure that all the institutions members participate and play an active and productive role in the education mechanism of the institution. (39)	0,5	100	0,825
	I attach importance to studies that increase cooperation in the institution. (45)	0,5		
Evaluation	I evaluate teachers objectively and equally. (57)	0,5		0,793

Note: The numbers in the table indicate the order of the question statements in the questionnaire.

*Managerial processes determining effective special education institution management in relation to special education teachers;* Based on the results of factor analysis of managerial processes, the following variables were used for regression analysis:

To proceed with testing the hypothesis. In the factor analysis for each element used in the model, the following Cronbach’s Alpha values were obtained and are shown in Table 5:

***Managerial processes determining effective special education institution management in relation to special education managers;*** Based on the results of factor analysis of managerial processes, the following variables were used for regression analysis:

To proceed with testing the hypothesis. In the factor analysis for each element used in the model, the following Cronbach’s Alpha values were obtained and are shown in Table 9:

**Table 9:** Measure of factor analysis applicability of north Cyprus, Turkey, and the UK.

			North Cyprus	Turkey	UK
Measure of Factor Analysis Applicability	Kaiser-Meyer-Olkin Measure of Sampling Adequacy		0,814	0,859	0,500
	Bartlett Test of Sphericity	Chi-Square df significance	44,590	207,266	4,123
			6	15	1
			0,000	0000	0,042

After the factor analysis, following Cronbach’s Alpha values for each element, the formation of our model and hypothesis were determined, and we proceeded with regression analyses.

#### 2.5.3 The model was created in line with the research findings in the study regarding effective institution management to be applied in special education institutions

The following hypothesis were formed with the model above (Fig. 1). Adhering to the aims of the research with the aforementioned tests, the hypothesis was tested.

Hypothesis 1 There is a positive relationship between the managerial processes against effective special education institution management, according to special education teachers’ opinions and demographic structures.

Results from Table 10 imply that, based on the opinions and demographic structures of special education teachers in North Cyprus, planning, organization, communication, and evaluation processes are positively associated with effective special education institution management and negatively with decision-making process, and none with influencing process. In Turkey, decision-making, organization, communication, and influence processes positively reinforce effective special education institution management. Planning process has a negative impact, and evaluation process has no impact at all. In the UK, it is found that communication and influencing processes are positively associated with effective special education institution management. However, decision-making, planning, organization, and evaluation processes have no impact at all on effective management. It could be deduced that based on the outcomes of the teachers’ opinions and their demographic structures in the conducted survey and the results of the regression analysis, planning, organization, communication, and evaluation processes have a greater impact in determining effective management than decision-making and influencing processes in special education institutions in North Cyprus. In Turkey, decision-making, organization, communication, and influencing processes have a greater impact on special education institution management than planning and evaluation processes. In the UK, communication and influencing processes are more important than decision-making, planning, organization, and evaluation in determining effective special education institution management. It is also evident that the communication process based on the teachers’ opinions and demographic structures determines the effective management of special education institutions in all countries.

**Table 10:** Regression analysis results of Hypothesis 1.

Managerial Processes	Effective Special Education Institution Management (Coordination)														
	North Cyprus					Turkey					UK				
	R^2^	F	ß	t	p	R^2^	F	ß	t	p	R^2^	F	ß	t	p
Decision-making	0,439	37,546	−0,048	6,128	0,000	0,577	79,179	0,261	8,898	0,000					
Planning	0,641	85,540	0,061	9,249	0,000	0,627	97,314	−0,113	9,865	0,000					
Organization	0,890	387,938	0,615	19,696	0,000	0,837	297,827	0,716	17,258	0,000					
Communication	0,812	206,763	0,005	14,379	0,000	0,825	273,823	0,145	16,548	0,000	0,873	124,220	0,337	11,145	0,000
Influencing						0,559	73,446	0,186	8,570	0,000	0,889	143,649	0,601	11,985	0,000
Evaluation	0,863	302,792	0,439	17,401	0,000										
Number of observations 130															

**Notes:** All sample mean institutional management score has been determined by the managerial processes. The weights are as follows: **For North Cyprus**-Institutional management score = −0,372 (constant term) - 0,048 (Unstandardized Betas for decision-making) + 0,061 (Unstandardized Betas for planning) + 0,615 (Unstandardized Betas for organization) + 0,005 (Unstandardized Betas for communication) + 0,439 (Unstandardized Betas for evaluation) + 0,245 (error term); **For Turkey**-Institutional management score = -0,768 (constant term) + 0,261(Unstandardized Betas for decision-making) - 0,113 (Unstandardized Betas for planning) + 0,716 (Unstandardized Betas for organization) + 0,145 (Unstandardized Betas for communication) + 0,186 (Unstandardized Betas for influencing) + 0,431 (error term); **For the UK**-Institutional management score = 0,259 (constant term) + 0,337 (Unstandardized Betas for communication) + 0,601 (Unstandardized Betas for influencing) + 0,281 (error term). The p values below 0.05 indicate significance at 10%, 5%, and 1%, respectively.

Hypothesis 2 There is a positive relationship between the managerial processes against effective special education institution management, according to special education institution managers’ opinions and demographic structures.

Results of Table 11 imply that, based on the opinions and demographic structures of managers in North Cyprus, the evaluation process is more influential than other managerial processes in the model in determining effective special education institution management and is significantly positive. In Turkey, it was found that managers' efforts to provide effective management in their special education institutions' decision-making and organization processes are more influential than other managerial processes in the model in determining effective special education institution management. In the UK, based on managers’ opinions like North Cyprus, the evaluation process is more influential in determining effective management in special education institutions. Moreover, Hypotheses 3 and 4 are generated to make the model robust and to determine which management processes are effective in determining effective special education institution management generally. Table 12 shows the regression results for Hypothesis 3 regarding teachers' opinions and demographic structures.

**Table 11:** Regression analysis results of Hypothesis 2.

Managerial Processes	Effective Special Education Institution Management (Coordination)														
	North Cyprus					Turkey					UK				
	R^2^	F	ß	t	p	R^2^	F	ß	t	p	R^2^	F	ß	t	p
Decision-making						0,420	20,292	0,181	4,505	0,000					
Organization						0,508	28,884	0,895	5,374	0,000					
Evaluation	0,839	41,822	0,812	6,467	0,000						0,167	4,626	0,582	2,151	0,042
Number of observations 45															

**Notes:** All sample mean institutional management score has been determined by the managerial processes based on the factor analysis in the study. The weights are as follows: **For North Cyprus**-Institutional management score = 0,853 (constant term) + 0,812 (Unstandardized Betas for evaluation) + 0,292 (error term); **For Turkey**-Institutional management score = −0,282 (constant term) + 0,181(Unstandardized Betas for decision-making) + 0,895 (Unstandardized Betas for organization) + 0,422 (error term); **For the UK**-Institutional management score = 1,405 (constant term) + 0,582 (Unstandardized Betas for evaluation) + 0,337 (error term). The p values below 0.05 indicate significance at 10%, 5%, and 1%, respectively.

**Table 12:** Regression analysis results of Hypothesis 3.

Managerial Processes	Effective Special Education Institution Management (Coordination) (North Cyprus, Turkey and the UK)				
	R^2^	F	ß	t	p
Decision-making	0,600	192,130	0,929	13,861	0,000
Planning	0,693	289,602	1,000	17,018	0,000
Organization	0,853	744,111	1,021	27,278	0,000
Communication	0,827	611,933	0,941	24,737	0,000
Influencing	0,639	226,641	0,961	15,055	0,000
Evaluation	0,792	488,065	0,909	22,092	0,000
Number of observations 130					

**Notes:** All sample mean institutional management score has been determined by the managerial processes. The weights are as follows: Institutional management score = −0,391 (constant term) + 0,929 (Unstandardized Betas for decision-making) + 1,000 (Unstandardized Betas for planning) + 1,021 (Unstandardized Betas for organization) + 0,941 (Unstandardized Betas for communication) + 0,961 (Unstandardized Betas for influencing) + 0,909 (Unstandardized Betas for evaluation) + 0,325 (error term. The p values below 0.05 indicate significance at 10%, 5%, and 1%, respectively.

Hypothesis 3 Managerial processes of decision-making, communication, organization, evaluation, planning, and influencing have an impact on effective special education institution management when teachers are concerned in North Cyprus, Turkey, and the UK.

Table 12 illustrates the regression results of hypothesis 3 where all independent variables' p values are below 0.05 and are strongly significant in determining effective special education institution management in all countries. The values of R^2^ determine the movements of the dependent variable, which are completely explained by the movements of the independent variable. Therefore, organization, communication, and evaluation processes have more impact on effective special education institution management when teachers are concerned in all countries.

Hypothesis 4 Managerial processes of decision-making, organization, and evaluation have more impact on effective special education institution management when managers are concerned in North Cyprus, Turkey, and the UK.

Table 13 illustrates the regression results of hypothesis 4 where independent variables (decision-making, organization, and evaluation) have p values below 0.05 and are strongly significant in determining effective special education institution management in all countries. The values of R^2^ determine the movements of the dependent variable which are completely explained by the movements in the independent variable. R^2^ also shows the explained variance rate and indicates the explanatory power between the variables [49]. As a result, when managers are involved in all countries, organization process have a greater impact on effective special education institution management. On the other hand, when the p value corresponding to the F statistic is less than 0.05, the H_0_ hypothesis is rejected. This means that estimates between variables are statistically possible. After a general ranking of the regression model is made with the F test, the significance test for each of the coefficients that make up the model is done with the t statistic. The t values in the tables indicate this statistic. The coefficients that failed the significance test were removed from the model, and regression analysis was performed. Thus, it is ensured that all parts of the model make a significant contribution to the explanation of the dependent variable [49].

**Table 13:** Regression analysis results of hypothesis 4.

Managerial Processes	Effective Special Education Institution Management (Coordination) (North Cyprus, Turkey and the UK)				
	R^2^	F	ß	t	p
Decision-making	0,334	21,568	0,523	2,410	0,000
Organization	0,603	65,217	1112	8,076	0,000
Evaluation	0,528	16,654	0,506	4,081	0,000
Number of observations 45					

**Notes:** All sample mean institutional management score has been determined by the managerial processes based on the factor analysis in the study. The weights are as follows: Institutional management score = −0,746 (constant term) + 0,523 (Unstandardized Betas for decision-making) + 1,112 (Unstandardized Betas for organization) + 0,506 (Unstandardized Betas for evaluation) + 0,362 (error term). The p values below 0.05 indicate significance at 10%, 5%, and 1% respectively.

## 3 Results

### 3.1 The results of Hypothesis 1

According to the empirical results of hypothesis 1, planning, organization, communication, and evaluation have a positive and significant effect and have a greater impact on effective institution management than decision-making and influencing processes, according to the demographic structures and views of special education teachers in North Cyprus. Altinay, Dagli and Altinay found that planning, organizing, decision-making, coordination, evaluation, and supervision were applied with a positive impact on the management processes in special education institutions in North Cyprus [50]. However, Abbasoğlu and Açıl emphasized that there are significant institutional deficiencies in the field of special education in North Cyprus, raising awareness about these deficiencies and stating that new studies are needed [[51], [52]]. In a similar study, Mchatton, Boyer, Shaunessy, Terry, and Farmer found that there are gaps in special education schools' management [53]. In addition, according to teachers’ views and demographic structures, decision-making, organization, communication, and influencing have a positive and significant effect on effective institution management rather than planning and evaluation management processes in Turkey. Girgin states that education managers rarely meet teacher expectations in terms of managerial processes in decision-making, planning, influence, communication, and evaluation processes; the findings, which are often met in the coordination and organization processes, have been reached. In the UK, communication and influencing are positively associated with effective institution management [54]. Decision-making, planning, organization, and evaluation, on the other hand, have no effect on effective management. It is also clear that in all countries, the communication process based on teachers' views and demographic structures determines effective institution management. Deliceırmak, in his research, found it “sufficient” in the decision-making, planning, organization, influence, coordination, and evaluation of the managerial processes, and "very" in the communication process [55]. The *p* values below 0.05 indicate significance at 10%, 5%, and 1%, respectively, for all parameters that the null hypothesis has been rejected.

### 3.2 The results of Hypothesis 2

The demographic structures and opinions of managers in North Cyprus support the empirical findings of hypothesis 2 that the evaluation process has a substantially greater impact than the other managerial processes in the model. In Turkey, it was found that managers' efforts at providing effective management in their special education institutions’ decision-making and organization processes are more influential than other managerial processes in the model. In the UK, the evaluation process is more influential in determining effective management in special education institutions. With our results in North Cyprus and the UK, like Büte's study shows us, managers said evaluation progress is important for managing the institutions, but at the same time, there are some problems with evaluation progress when executing managerial processes [56]. Several issues occurred throughout the evaluation process as a result of the lack of inspectors who handled themselves professionally. In another study, İşman found that due to inadequate evaluations, the quality of the education services provided had reduced [4]. As a result of their research, Kosir and Jezovsek stated that the evaluation process of the managerial processes was not carried out deliberately and correctly [57]. In addition, the research findings showed that school management was in favor of creating awareness at the stage of creating school culture. Empirical results show that in all countries, efficient special education institution management is determined by the decision-making, organization, and evaluation processes based on managers' viewpoints and demographic structures. The *p* values below 0.05 indicate significance at 10%, 5%, and 1%, respectively, for all parameters that the null hypothesis has been rejected.

### 3.3 The results of Hypothesis 3

According to the empirical results of hypothesis 3, managerial processes of decision-making, communication, organization, evaluation, planning, and influencing have an impact on effective special education institution management when teachers are concerned in North Cyprus, Turkey, and the UK. Results of hypothesis 3 shows us that all independent variables' *p* values are below 0.05 and are strongly significant in determining effective special education institution management in the model.

### 3.4 The results of Hypothesis 4

The results of hypothesis 4 imply that when managers are involved, managerial processes of decision-making, organization, and evaluation have an impact on effective special education institution management in North Cyprus, Turkey, and the UK. The findings also demonstrate that all independent variables, which determine the effective management of special education institutions in all countries, have *p* values that are less than 0.05 and are strongly significant. Based on a similar study by Lashley and Boscardin, in their research, the education managers working in special education schools needed in-service training programs in the field of special education due to some inadequacies, and it was extremely important for the managers of both special education schools and general education schools to work together [58]. As a result of their research, standards for effective management have been developed. According to Ağdelen, however, it has been suggested that school managers should be prepared before service with educational models based on scientific methods and that they should be developed on the job [59].

The purpose of the study was to evaluate whether managerial processes affect how well special education institutions in three countries are managed. The model developed for the study served as the foundation for the hypothesis, which revealed that all managerial processes are positively and strongly related to effective management from the teachers' perspective. In contrast, according to managers in all countries, decision-making, organization, and evaluation are strongly and favorably related to effective management. Planning, organization, communication, and evaluation processes have a greater impact on determining effective management than decision-making and influencing in special education institutions in North Cyprus, according to the results of the regression analysis based on the opinions of the teachers and their demographic structures. Planning and evaluation processes have less impact on the management of special education institutions in Turkey than do decision-making, organization, communication, and influencing processes. In the UK, decision-making, planning, organization, and evaluation were less significant factors in predicting effective management of special education institutions than communication and the ability to influence processes. It is also obvious that the management of special education institutions in all nation is determined by the communication process based on the views of the teachers. The demographic structures and views of the managers in North Cyprus' institutions lend credence to empirical findings, which show that the evaluation process has a far bigger effect than the other managerial processes in the model. It was discovered in Turkey that managers who provide effective management in their special education institutions have a greater impact on decision-making and organization processes than other managerial processes in the model. The evaluation process has a greater impact on effective management in special education institutions in the UK. In contrast, according to managers in all countries, decision-making, organization, and evaluation are strongly and favorably related to effective management. Planning, organization, communication, and evaluation positively determine effective special education institution management, whereas decision-making and lack of influence negatively determine it, according to the views of special education teachers in North Cyprus.

## 4 Discussion

Selvi, in her research titled "Analysis of Managerial Problems in Special Education Institutions" investigated the managerial processes applied in special education institutions and developed solutions for the problems that may arise [5]. The sample for the research consisted of education managers, teachers, and parents working in public special education institutions and rehabilitation centers. The results of the research show that special education institutions and rehabilitation centers are not managed professionally, the personal rights of teachers working in rehabilitation centers are limited, and special education practices are not properly supervised during the inspections. Nevertheless, there have been studies on managerial processes applied in pre-school and primary education institutions in the last 20 years, the lack of studies on managerial processes applied in special education institutions makes this research important. Therefore, the essence of this study involves examining the relationship between managerial processes and effective special education institution management. To carry out the research, a survey was conducted. The chosen sample consists of special education teachers and managers working in special education institutions in North Cyprus, Turkey, and the UK. The parameters that make up the managerial processes are decision-making, planning, organization, communication, influencing, and evaluation. The coordination parameter, on the other hand, defines effective special education institution management as an independent variable in the constructed model. Factor analyses were conducted to determine the accuracy and reliability of the questions asked about the managerial processes in the survey. The effect of managerial processes on effective special education institution management was calculated by making regression analyses of the parameters that make up the model, provided that the alpha value determined for each question is 0.70 and above in the factor analysis.

The research examined the relationship between independent variables and dependent variables using the model created by calculating the averages after selecting the questions representing the managerial processes based on factor analysis. According to Bursalıoğlu, coordination is important for effective management, and successful corporate managers perform the duties of creating an appropriate organizational climate, selecting and developing talented subordinates, and developing integrated plans and programs that their subordinates can implement, leading to an effective management system [34]. The researchers are said to be capable of meeting the requirements [20] and coordination allows educational institutions to be more effective in achieving their goals. As discussed above and in the previous sections, organization process can establish effective management if it can coordinate the organizational climate appropriately. Therefore, the coordination is consistent with effective management that is influenced by maintaining decent decision-making, planning, organization, communication, influencing, and evaluation in educational institutions. Tofur and Yıldırım said that, there are numerous factors that contribute to educational success, and one of the most significant is the effective use of management processes by the education manager. And found that, influencing is a difficult task to motivate individuals and achieve goals [60].

Erden and Erden, in their research titled "Current Problems in North Cyprus Education System" aimed to determine the views of the managers and teachers working in primary schools, secondary education, and technical education in the North Cyprus education system on the problems of the education system [61]. They found that the issues closely related to the managerial processes applied in educational institutions, such as political reasons, lack of physical structure and equipment, school finance, training programs, inspections, in-service training of teachers, and managerial problems, constitute current problems in the education system. On the other hand, Roberts examined the perceptions of school managers and teachers working in special education institutions [23]. He stated that the responsibilities specified in the IDEA (Individualized Education Act for Disabled Students) regarding the education management in special education institutions were not fulfilled by the school managers, and an intervention preparation program was proposed. Therefore, it is recommended that special education institution managers should apply the model resulting from our research in the study.

The effect of the managerial processes that emerged in this research on the effective management used in special education institutions can be strengthened by expanding the number of participants from different countries in subsequent research and by collecting data from parents as participants in similar studies. This research is limited to the data collected from special education teachers, school managers, and the data collected from special education institutions in three countries (North Cyprus, Turkey, and the UK) that comprise the universe of the research. In future studies, it is considered that collecting data from parents, special education teachers, and school managers working in special education institutions operating more different countries will eliminate such limitations and strengthen the further research. Practical contribution of this work is it enables educational policymakers, school managers, teachers, researchers, and the community to comprehend the changing trends in school management to maximise student outcomes holistically. It also points out the gaps in the existing literature so that future researchers will have a good set of directions for reference. In the same vein, this framework contributes theoretically and conceptually and would guide future work to further expand the existing theories by involving multiple domains related to special education school management.

The name of the ethics committee and date of approval of the report is as follow: The project proposal has been approved by the “Scientific Research Ethics Committee” of the Near East University, project number NDI/EB/2020/427, on the August 26, 2020.

## Author contribution statement

KAZIM KÜÇÜKALKAN: Conceived and designed the experiments; Performed the experiments; Analyzed and interpreted the data; Contributed reagents, materials, analysis tools or data; Wrote the paper.

Mustafa AVCİN: Analyzed and interpreted the data; Wrote the paper.

Mukaddes SAKALLI DEMİROK: Conceived and designed the experiments.

## Data availability statement

Data will be made available on request

## Additional information

No additional information is available for this paper

## Declaration of competing interest

The authors declare that they have no known competing financial interests or personal relationships that could have appeared to influence the work reported in this paper.
