# Supplementary material for: Assessment of managerial processes applied in multinational special education institutions
Source: Heliyon. 2023 Aug 30;9(9):e19514. doi: 10.1016/j.heliyon.2023.e19514 (PMC10558714; doi:10.1016/j.heliyon.2023.e19514)
Supplement: Multimedia component 1 [file mmc1.docx]

Dear Participant,

This questionnaire was prepared for the doctoral thesis named “Assessment of Managerial Processes Applied in Multinational Special Education Institutions”.

The questionnaire consists of 2 parts. The first part includes questions containing personal and occupational information; the second part, you will find entries related to managerial processes of institution managers (decision-making, planning, organization, communication, coordination, influence and evaluation) which includes 5-point likert type questions. Check the best option for you. Please answer all of the entries and do not forget to choose only one option for each of them.

Achieving reliable results for the research depends to your clear, accurate and honest answers. Estimated response time is 10-15 minutes. Please read the questions carefully and mark the best option by putting an “X” into the box. You don’t need to write your name and surname on the questionnaire.

Your answers will only be used for academic purposes and will never be shared with third parties. Please remember that obtaining reliable results is possible only by answering all of the questions. I would like to thank and give you my best regards for taking your precious time and consideration and for your contribution to my research.

Associate Prof. Mukaddes SAKALLI DEMİROK Kazım KÜÇÜKALKAN

Thesis Advisor PhD Student

***For Institution Managers/******Assistant Managers***

**PART I- Personal Information’s**

1. **Gender**

( ) Female ( ) Male

1. **Age**

( )20-25 ( )26-30 ( )31-35 ( )36-40 ( )41<

1. **Graduation**

( ) Associate Degree ( ) Bachelor’s Degree ( ) MBA ( ) PhD ( ) Other

1. **Occupational Seniority (For Institution Managers/Assistant Managers)**

( ) 0-5 year ( ) 6-10 year ( ) 11-15 year ( ) 16-20 year ( ) 21<

1. **Your duty at the Special Education Institution**

( ) Institution Manager ( ) Assistant Manager ( ) Other (……………………………………..)

| **PART II: Management Processes**  **(5-Point Likert Type Questions)** | **(1)**  **Never** | | | **(2)**  **Very Rare** | | | | | **(3)**  **Occasionally** | | | | | | **(4)**  **Mostly** | | | | | | **(5)**  **Always** | | | | | |  |
| --- | --- | --- | --- | --- | --- | --- | --- | --- | --- | --- | --- | --- | --- | --- | --- | --- | --- | --- | --- | --- | --- | --- | --- | --- | --- | --- | --- |
| **A. Decision-Making** |  | | |  | | | | |  | | | | | |  | | | | | |  | | | | | |  |
| I make decisions after meeting with both parents and staff to solve institutional problems. | ***(*** | | ***)*** | ***(*** | | | ***)*** | | ***(*** | | ***)*** | | | |  | | ***( )*** | | | | ***(*** | | ***( )*** | | | | |
| During decision making I ensure that authority and responsibilities are distributed equally and in a balanced manner. | ***(*** | | ***)*** | ***(*** | | | ***)*** | | ***(*** | | ***)*** | | | |  | | ***( )*** | | | | ***(*** | | ***( )*** | | | | |
| I make decisions about education and training (monthly, daily plan, parents meeting, family education etc.) together with teachers. | ***(*** | | ***)*** | ***(*** | | | ***)*** | | ***(*** | | ***)*** | | | |  | | ***( )*** | | | | ***(*** | | ***( )*** | | | | |
| I consult with staff in order to obtain beneficial sources of information from relevant publications and people. | ***(*** | | ***)*** | ***(*** | | | ***)*** | | ***(*** | | ***)*** | | | |  | | ***( )*** | | | | ***(*** | | ***( )*** | | | | |
| I make decisions together with staff for determining, regulating and supporting productive usage of equipment and fixtures in line with the needs of the institution. | ***(*** | | ***)*** | ***(*** | | | ***)*** | | ***(*** | | ***)*** | | | |  | | ***( )*** | | | | ***(*** | | ***( )*** | | | | |
| During the decision-making process I behave in a democratic and participatory manner. | ***(*** | | ***)*** | ***(*** | | | ***)*** | | ***(*** | | ***)*** | | | |  | | ***( )*** | | | | ***(*** | | ***( )*** | | | | |
| I obtain staff opinion on the revenue and expenditure of institutional budgets. | ***(*** | | ***)*** | ***(*** | | | ***)*** | | ***(*** | | ***)*** | | | |  | | ***( )*** | | | | ***(*** | | ***( )*** | | | | |
| I benefit from both staff and experts for topics relating to an institution. | ***(*** | | ***)*** | ***(*** | | | ***)*** | | ***(*** | | ***)*** | | | |  | | ***( )*** | | | | ***(*** | | ***( )*** | | | | |
| I ensure all staff attend meetings in relation to decisions taken about the institution. | ***(*** | | ***)*** | ***(*** | | | ***)*** | | ***(*** | | ***)*** | | | |  | | ***( )*** | | | | ***(*** | | ***( )*** | | | | |
| I assign authorization to my subordinates while making a decision. | ***(*** | | ***)*** | ***(*** | | | ***)*** | | ***(*** | | ***)*** | | | |  | | ***( )*** | | | | ***(*** | | ***( )*** | | | | |
|  | **(1)**  **Never** | | | **(2)**  **Very Rare** | | | | | **(3)**  **Occasionally** | | | | | | **(4)**  **Mostly** | | | | | | **(5)**  **Always** | | | | | |  |
| **B. Planning** |  | | |  | | | | |  | | | | | |  | | | | | |  | | | | | |  |
| I plan education and training activities for the current education year (monthly, daily plans, parents meeting, family educations, celebrating special days etc.) with teachers. | ***( )*** | |  | ***(*** | | | ***)*** | | ***(*** | | ***)*** | | | |  | | | ***( )*** | | | ***(*** | | ***( )*** | | | | |
| I guide teachers in the preparation and compatibility of educational plans. | ***( )*** | |  | ***(*** | | | ***)*** | | ***(*** | | ***)*** | | | |  | | | ***( )*** | | | ***(*** | | ***( )*** | | | | |
| I take into consideration staff opinions while planning cultural activities and sightseeing trips. | ***( )*** | |  | ***(*** | | | ***)*** | | ***(*** | | ***)*** | | | |  | | | ***( )*** | | | ***(*** | | ***( )*** | | | | |
| I plan the institutions budget, its revenues and the expenses to be made as a result of joint decisions taken at teacher’s council meeting. | ***( )*** | |  | ***(*** | | | ***)*** | | ***(*** | | ***)*** | | | |  | | | ***( )*** | | | ***(*** | | ***( )*** | | | | |
| I make plans about the institution in collaboration with teachers and employees. | ***( )*** | | | ***( )*** | | | | | ***( )*** | | | | | | ***( )*** | | | | | | ***( )*** | | | | | |  |
| I take into consideration environmental expectations and opportunities while planning educational activities. | ***( )*** | | | ***( )*** | | | | | ***( )*** | | | | | | ***( )*** | | | | | | ***( )*** | | | | | |  |
| I ensure that student registration and transfer related procedures (for qualified students) are carried out properly. | ***( )*** | | | ***( )*** | | | | | ***( )*** | | | | | | ***( )*** | | | | | | ***( )*** | | | | | |  |
| I plan the supply, distribution, usage and protection of the institution’s teaching equipment. | ***( )*** | | | ***( )*** | | | | | ***( )*** | | | | | | ***( )*** | | | | | | ***( )*** | | | | | |  |
| I ensure that all the precautions relating to the cleanliness and order of the institution are taken. | ***( )*** | | | ***( )*** | | | | | ***( )*** | | | | | | ***( )*** | | | | | | ***( )*** | | | | | |  |
| I plan schedules regarding families (parent meeting dates, family education seminars etc.) at the teacher’s council meeting. | ***( )*** | | | ***( )*** | | | | | ***( )*** | | | | | | ***( )*** | | | | | | ***( )*** | | | | | |  |
|  | **(1)**  **Never** | | | **(2)**  **Very Rare** | | | | | **(3)**  **Occasionally** | | | | | | **(4)**  **Mostly** | | | | | | **(5)**  **Always** | | | | | |  |
| **C. Organization** |  | | |  | | | | |  | | | | | |  | | | | | |  | | | | | |  |
| I explain duties, roles and responsibilities of the institution to staff (teachers and other employees) in a written and clear way. | ***(*** | ***)*** | | ***(*** | | | | ***)*** | ***(*** | | | ***)*** | | |  | | | | ***( )*** | |  | | | ***( )*** | | |  |
| While assigning a task I pay attention to prevent any conflict of roles. | ***(*** | ***)*** | | ***(*** | | | | ***)*** | ***(*** | | | ***)*** | | |  | | | | ***( )*** | |  | | | ***( )*** | | |  |
| I ensure that all institutional related documents are processed in time and announced to institutional employees. | ***(*** | ***)*** | | ***(*** | | | | ***)*** | ***(*** | | | ***)*** | | |  | | | | ***( )*** | |  | | | ***( )*** | | |  |
| There is a division of labor between teachers and other staff. | ***(*** | ***)*** | | ***(*** | | | | ***)*** | ***(*** | | | ***)*** | | |  | | | | ***( )*** | |  | | | ***( )*** | | |  |
| I organize the institution appropriately, in consideration and parallel to the institutions goals and objectives. | ***(*** | ***)*** | | ***(*** | | | | ***)*** | ***(*** | | | ***)*** | | |  | | | | ***( )*** | |  | | | ***( )*** | | |  |
| I plan the institutions activities in compliance with effective usage of both human and pecuniary resources. | ***(*** | ***)*** | | ***(*** | | | | ***)*** | ***(*** | | | ***)*** | | |  | | | | ***( )*** | |  | | | ***( )*** | | |  |
| By building good relations with the environment I benefit from both people and groups in parallel with an institutions aims. | ***(*** | ***)*** | | ***(*** | | | | ***)*** | ***(*** | | | ***)*** | | |  | | | | ***( )*** | |  | | | ***( )*** | | |  |
| I frequently gather teacher’s council to find solutions regarding teaching and education in parallel with institution objectives. | ***(*** | ***)*** | | ***(*** | | | | ***)*** | ***(*** | | | ***)*** | | |  | | | | ***( )*** | |  | | | ***( )*** | | |  |
|  | **(1)**  **Never** | | | **(2)**  **Very Rare** | | | | | **(3)**  **Occasionally** | | | | | | **(4)**  **Mostly** | | | | | | **(5)**  **Always** | | | | | |  |
| **D. Communication** |  | | |  | | | | |  | | | | | |  | | | | | |  | | | | | |  |
| I benefit from clear and effective communication methods and tools while communicating with teachers. | ***( )*** | | | ***)*** | ***(*** | | ***)*** | | | ***(*** | | | | ***)*** | |  | | | | ***( )*** | |  | | | | ***( )*** | |
| I do work for the development of communication between staff. | ***( )*** | | | ***)*** | ***(*** | | ***)*** | | | ***(*** | | | | ***)*** | |  | | | | ***( )*** | |  | | | | ***( )*** | |
| I promptly notify teachers of any change in the legislations and application. | ***( )*** | | | ***)*** | ***(*** | | ***)*** | | | ***(*** | | | | ***)*** | |  | | | | ***( )*** | |  | | | | ***( )*** | |
| I inform teachers regarding newly received directions and procedures. | ***( )*** | | | ***)*** | ***(*** | | ***)*** | | | ***(*** | | | | ***)*** | |  | | | | ***( )*** | |  | | | | ***( )*** | |
| I use vertical and horizontal communication types in the institution. | ***( )*** | | | ***)*** | ***(*** | | ***)*** | | | ***(*** | | | | ***)*** | |  | | | | ***( )*** | |  | | | | ***( )*** | |
| I ensure that a warm and friendly atmosphere is created between staff relations within the institution. | ***( )*** | | | ***)*** | ***(*** | | ***)*** | | | ***(*** | | | | ***)*** | |  | | | | ***( )*** | |  | | | | ***( )*** | |
| I follow the course of any activities organized in the institution and consult with teachers about any observed deficiencies and measures to be taken. | ***( )*** | | | ***)*** | ***(*** | | ***)*** | | | ***(*** | | | | ***)*** | |  | | | | ***( )*** | |  | | | | ***( )*** | |
| I allow teachers to express their ideas and opinions clearly within the institution. | ***( )*** | | | ***)*** | ***(*** | | ***)*** | | | ***(*** | | | | ***)*** | |  | | | | ***( )*** | |  | | | | ***( )*** | |
| I ensure that the wishes and complaints of staff in charge of teachers are easily conveyed to the institutions management. | ***( )*** | | | ***)*** | ***(*** | | ***)*** | | | ***(*** | | | | ***)*** | |  | | | | ***( )*** | |  | | | | ***( )*** | |
| I benefit from clear and effective communication methods and tools while communicating with teachers. | ***( )*** | | |  | ***(*** | | ***)*** | | | ***(*** | | | | ***)*** | |  | | | | ***( )*** | |  | | | | ***( )*** | |
| **E. Coordination** |  | | |  | | | | |  | | | | | |  | | | | | |  | | | | | |  |
|  | **(1)**  **Never** | | | **(2)**  **Very Rare** | | | | | **(3)**  **Occasionally** | | | | | | **(4)**  **Mostly** | | | | | | **(5)**  **Always** | | | | | |  |
| I ensure that all the institutions members participate and play an active and productive role in the education mechanism of the institution. | ***( )*** | | |  | ***( )*** | |  | | | ***( )*** | | | |  | | ***(*** | | | | ***)*** | |  | | | | ***( )*** | |
| I encourage cooperation between teachers, other employees, students, parents, school council and top-level managers to assist in the institution reaching its goals. | ***( )*** | | |  | ***( )*** | |  | | | ***( )*** | | | |  | | ***(*** | | | | ***)*** | |  | | | | ***( )*** | |
| I schedule educational activities for teachers (monthly-daily plans, parent meetings, family education and celebrating special days) and ensure that all the activities are carried out collaboratively. | ***( )*** | | |  | ***( )*** | |  | | | ***( )*** | | | |  | | ***(*** | | | | ***)*** | |  | | | | ***( )*** | |
| I prioritize in-service education and ensure that all the staff members are trained well in this field. | ***( )*** | | |  | ***( )*** | |  | | | ***( )*** | | | |  | |  | | | | ***( )*** | |  | | | | ***( )*** | |
| I play a conciliatory role between teachers, top-level managers (ex. director of national education) students, parents and institution staff. | ***( )*** | | |  | ***( )*** | |  | | | ***( )*** | | | |  | |  | | | | ***( )*** | |  | | | | ***( )*** | |
| I inform institution employees regarding each other’s work. | ***( )*** | | |  | ***( )*** | |  | | | ***( )*** | | | |  | |  | | | | ***( )*** | |  | | | | ***( )*** | |
| I attach importance to studies that increase cooperation in the institution. | ***( )*** | | |  | ***( )*** | |  | | | ***( )*** | | | |  | |  | | | | ***( )*** | |  | | | | ***( )*** | |
|  | **(1)**  **Never** | | | **(2)**  **Very Rare** | | | | | **(3)**  **Occasionally** | | | | | | **(4)**  **Mostly** | | | | | | **(5)**  **Always** | | | | | |  |
| **F. Influence** |  | | |  | | | | |  | | | | | |  | | | | | |  | | | | | |  |
| I use reward as a motivation source to boost/increase teachers’ success. | ***( )*** | | | ***)*** | ***( )*** | |  | | | ***( )*** | | | |  | | ***(*** | | | | ***)*** | |  | | | | ***( )*** | |
| I use menace and punishment to motivate teachers. | ***( )*** | | | ***)*** | ***( )*** | |  | | | ***( )*** | | | |  | | ***(*** | | | | ***)*** | |  | | | | ***( )*** | |
| I use both material and moral rewarding to motivate teachers. | ***( )*** | | | ***)*** | ***( )*** | |  | | | ***( )*** | | | |  | | ***(*** | | | | ***)*** | |  | | | | ***( )*** | |
| I assist teachers and other employees about embracing their work and fully committing themselves to the institution. | ***( )*** | | |  | ***( )*** | |  | | | ***( )*** | | | |  | | ***(*** | | | | ***)*** | |  | | | | ***( )*** | |
| I find immediate and effective solutions to the problems experienced within the institution. | ***( )*** | | |  | ***( )*** | |  | | | ***( )*** | | | |  | | ***(*** | | | | ***)*** | |  | | | | ***( )*** | |
| I motivate teachers for using various education methods and techniques. | ***( )*** | | |  | ***( )*** | |  | | | ***( )*** | | | |  | | ***(*** | | | | ***)*** | |  | | | | ***( )*** | |
| I refer to social and psychological influencing methods rather than using my authority. | ***( )*** | | |  | ***( )*** | |  | | | ***( )*** | | | |  | | ***(*** | | | | ***)*** | |  | | | | ***( )*** | |
|  | **(1)**  **Never** | | | **(2)**  **Very Rare** | | | | | **(3)**  **Occasionally** | | | | | | **(4)**  **Mostly** | | | | | | **(5)**  **Always** | | | | | |  |
| **G. Evaluation** |  | | |  | | | | |  | | | | | |  | | | | | |  | | | | | |  |
| I monitor and evaluate teacher’s lectures and work in terms of their efficiency at various times of the academic year. | ***( )*** | | |  | | ***( )*** |  | | ***( )*** | | | |  | | ***(*** | | | | | ***)*** |  | | | | ***( )*** | |  |
| I carry out evaluation with teachers and prepare regulations to keep the institution up-to-date modern and more effective. | ***( )*** | | |  | | ***( )*** |  | | ***( )*** | | | |  | | ***(*** | | | | | ***)*** |  | | | | ***( )*** | |  |
| I implement the results of inspections and evaluations together with teachers. | ***( )*** | | |  | | ***( )*** |  | | ***( )*** | | | |  | | ***(*** | | | | | ***)*** |  | | | | ***( )*** | |  |
| I oversee the time keeping of teacher’s scheduled activities, including the consistency and harmony of these activities. | ***( )*** | | |  | | ***( )*** |  | | ***( )*** | | | |  | |  | | | | | ***( )*** |  | | | | ***( )*** | |  |
| I evaluate teachers objectively and equally. | ***( )*** | | |  | | ***( )*** |  | | ***( )*** | | | |  | | ***(*** | | | | | ***)*** |  | | | | ***( )*** | |  |
| I display constructive and developing behavior when delivering results of an audit and its evaluation. | ***( )*** | | |  | | ***( )*** |  | | ***( )*** | | | |  | | ***(*** | | | | | ***)*** |  | | | | ***( )*** | |  |
| I see the institution as a whole and assess and evaluate the effectiveness and efficiency level of the school. | ***( )*** | | |  | | ***( )*** |  | | ***( )*** | | | |  | | ***(*** | | | | | ***)*** |  | | | | ***( )*** | |  |
| I make objective and motivating evaluations when evaluating the personnel of an institution. | ***( )*** | | |  | | ***( )*** |  | | ***( )*** | | | |  | | ***(*** | | | | | ***)*** |  | | | | ***( )*** | |  |
| I supervise teacher’s motivation to work efficiently. | ***( )*** | | | ***( )*** | | | | | ***( )*** | | | | | | ***( )*** | | | | | | ***( )*** | | | | | |  |

***Interview Date: ………………………………….***

***Interview Place: ……………………………......***

***Your Institutions’ Name: …………………………***

Dear Participant,

This questionnaire was prepared for the doctoral thesis named “Assessment of Managerial Processes Applied in Multinational Special Education Institutions”.

The questionnaire consists of 2 parts. The first part includes questions containing personal and occupational information; the second part, you will find entries related to managerial processes of institution managers (decision-making, planning, organization, communication, coordination, influence and evaluation) which includes 5-point likert type questions. Check the best option for you. Please answer all of the entries and do not forget to choose only one option for each of them.

Achieving reliable results for the research depends to your clear, accurate and honest answers. Estimated response time is 10-15 minutes. Please read the questions carefully and mark the best option by putting an “X” into the box. You don’t need to write your name and surname on the questionnaire.

Your answers will only be used for academic purposes and will never be shared with third parties. Please remember that obtaining reliable results is possible only by answering all of the questions. I would like to thank and give you my best regards for taking your precious time and consideration and for your contribution to my research.

Associate Prof. Mukaddes SAKALLI DEMİROK Kazım KÜÇÜKALKAN

Thesis Advisor PhD Student

***For Teachers***

**PART I- Personal Information**

1. **Gender**

( ) Female ( ) Male

1. **Age**

( )20-25 ( )26-30 ( )31-35 ( )36-40 ( )41<

1. **Graduation**

( ) Associate Degree ( ) Bachelor’s Degree ( ) MBA ( ) PhD ( ) Other

1. **Occupational Seniority**

( ) 0-5 year ( ) 6-10 year ( ) 11-15 year ( ) 16-20 year ( ) 21<

1. **Your duty at the Special Education Institution**

( ) Special Education Teacher ( ) Other (………………………………………..)

| **PART II: Management Processes**    **Your Institution Manager;** | **(1)**  **Never** | | | | **(2)**  **Very Rare** | | | | **(3)**  **Occasionally** | | | | **(4)**  **Mostly** | | | | **(5)**  **Always** | |  |
| --- | --- | --- | --- | --- | --- | --- | --- | --- | --- | --- | --- | --- | --- | --- | --- | --- | --- | --- | --- |
| **A. Decision-Making** |  | | | |  | | | |  | | | |  | | | |  | |  |
| Makes decisions after meeting with both parents and staff to solve institutional problems. | ***(*** | | | ***)*** | ***(*** | | | ***)*** | ***(*** | | | ***)*** | ***(*** | | | ***)*** | ***(*** | ***( )*** |  |
| During decision making he ensures that authority and responsibilities are distributed equally and in a balanced manner. | ***(*** | | | ***)*** | ***(*** | | | ***)*** | ***(*** | | | ***)*** | ***(*** | | | ***)*** | ***(*** | ***( )*** |  |
| Makes decisions about education and training (monthly, daily plan, parents meeting, family education etc.) together with teachers. (3) | ***(*** | | | ***)*** | ***(*** | | | ***)*** | ***(*** | | | ***)*** | ***(*** | | | ***)*** | ***(*** | ***( )*** |  |
| Consults with staff in order to obtain beneficial sources of information from relevant publications and people. | ***(*** | | | ***)*** | ***(*** | | | ***)*** | ***(*** | | | ***)*** | ***(*** | | | ***)*** | ***(*** | ***( )*** |  |
| Makes decisions together with staff for determining, regulating and supporting productive usage of equipment and fixtures in line with the needs of the institution. (5) | ***(*** | | | ***)*** | ***(*** | | | ***)*** | ***(*** | | | ***)*** | ***(*** | | | ***)*** | ***(*** | ***( )*** |  |
| During the decision-making process he behaves in a democratic and participatory manner. | ***(*** | | | ***)*** | ***(*** | | | ***)*** | ***(*** | | | ***)*** | ***(*** | | | ***)*** | ***(*** | ***( )*** |  |
| Obtains staff opinion on the revenue and expenditure of institutional budgets. | ***(*** | | | ***)*** | ***(*** | | | ***)*** | ***(*** | | | ***)*** | ***(*** | | | ***)*** | ***(*** | ***( )*** |  |
| Benefits from both staff and experts for topics relating to an institution. | ***(*** | | | ***)*** | ***(*** | | | ***)*** | ***(*** | | | ***)*** | ***(*** | | | ***)*** | ***(*** | ***( )*** |  |
| Ensures all staff attend meetings in relation to decisions taken about the institution. (9) | ***(*** | | | ***)*** | ***(*** | | | ***)*** | ***(*** | | | ***)*** | ***(*** | | | ***)*** | ***(*** | ***( )*** |  |
| Assigns authorization to him subordinates while making a decision. | ***(*** | | | ***)*** | ***(*** | | | ***)*** | ***(*** | | | ***)*** | ***(*** | | | ***)*** | ***(*** | ***( )*** |  |
| **Your Institution Manager;** | **(1)**  **Never** | | | | **(2)**  **Very Rare** | | | | **(3)**  **Occasionally** | | | | **(4)**  **Mostly** | | | | **(5)**  **Always** | |  |
| **B. Planning** |  | | | |  | | | |  | | | |  | | | |  | |  |
| Plans education and training activities for the current education year (monthly, daily plans, parents meeting, family educations, celebrating special days etc.) with teachers. (11) | ***(*** | | | ***)*** | ***(*** | | | ***)*** | ***(*** | | | ***)*** | ***(*** | | | ***)*** | ***(*** | ***( )*** |  |
| Guides teachers in the preparation and compatibility of educational plans. | ***(*** | | | ***)*** | ***(*** | | | ***)*** | ***(*** | | | ***)*** | ***(*** | | | ***)*** | ***(*** | ***( )*** |  |
| Takes into consideration staff opinions while planning cultural activities and sightseeing trips. (13) | ***(*** | | | ***)*** | ***(*** | | | ***)*** | ***(*** | | | ***)*** | ***(*** | | | ***)*** | ***(*** | ***( )*** |  |
| Plans the institutions budget, its revenues and the expenses to be made as a result of joint decisions taken at teacher’s council meeting. | ***( )*** | | | | ***( )*** | | | | ***( )*** | | | | ***( )*** | | | | ***( )*** | |  |
| Makes plans about the institution in collaboration with teachers and employees. | ***( )*** | | | | ***( )*** | | | | ***( )*** | | | | ***( )*** | | | | ***( )*** | |  |
| Takes into consideration environmental expectations and opportunities while planning educational activities. | ***( )*** | | | | ***( )*** | | | | ***( )*** | | | | ***( )*** | | | | ***( )*** | |  |
| Ensures that student registration and transfer related procedures (for qualified students) are carried out properly. | ***( )*** | | | | ***( )*** | | | | ***( )*** | | | | ***( )*** | | | | ***( )*** | |  |
| Plans the supply, distribution, usage and protection of the institution’s teaching equipment. | ***( )*** | | | | ***( )*** | | | | ***( )*** | | | | ***( )*** | | | | ***( )*** | |  |
| Ensures that all the precautions relating to the cleanliness and order of the institution are taken. | ***( )*** | | | | ***( )*** | | | | ***( )*** | | | | ***( )*** | | | | ***( )*** | |  |
| Plans schedules regarding families (parent meeting dates, family education seminars etc.) at the teacher’s council meeting. | ***( )*** | | | | ***( )*** | | | | ***( )*** | | | | ***( )*** | | | | ***( )*** | |  |
| **Your Institution Manager;** | **(1)**  **Never** | | | | **(2)**  **Very Rare** | | | | **(3)**  **Occasionally** | | | | **(4)**  **Mostly** | | | | **(5)**  **Always** | |  |
| **C. Organization** |  | | | |  | | | |  | | | |  | | | |  | |  |
| Explains duties, roles and responsibilities of the institution to staff (teachers and other employees) in a written and clear way. (21) | ***(*** | | ***)*** | | ***(*** | | ***)*** | | ***(*** | | ***)*** | | ***(*** | | ***)*** | | ***( )*** | | **)** |
| While assigning a task he pays attention to prevent any conflict of roles. | ***(*** | | ***)*** | | ***(*** | | ***)*** | | ***(*** | | ***)*** | | ***(*** | | ***)*** | | ***( )*** | |  |
| Ensures that all institutional related documents are processed in time and announced to institutional employees. (23) | ***(*** | | ***)*** | | ***(*** | | ***)*** | | ***(*** | | ***)*** | | ***(*** | | ***)*** | | ***( )*** | |  |
| There is a division of labor between teachers and other staff. (24) | ***(*** | | ***)*** | | ***(*** | | ***)*** | | ***(*** | | ***)*** | | ***(*** | | ***)*** | | ***( )*** | |  |
| Organizes the institution appropriately, in consideration and parallel to the institutions goals and objectives. | ***(*** | | ***)*** | | ***(*** | | ***)*** | | ***(*** | | ***)*** | | ***(*** | | ***)*** | | ***( )*** | |  |
| Plans the institutions activities in compliance with effective usage of both human and pecuniary resources. | ***(*** | | ***)*** | | ***(*** | | ***)*** | | ***(*** | | ***)*** | | ***(*** | | ***)*** | | ***( )*** | |  |
| By building good relations with the environment he benefit from both people and groups in parallel with an institutions aims. (27) | ***(*** | | ***)*** | | ***(*** | | ***)*** | | ***(*** | | ***)*** | | ***(*** | | ***)*** | | ***( )*** | |  |
| He frequently gathers teacher’s council to find solutions regarding teaching and education in parallel with institution objectives. | ***(*** | | ***)*** | | ***(*** | | ***)*** | | ***(*** | | ***)*** | | ***(*** | | ***)*** | | ***( )*** | |  |
| **Your Institution Manager;** | **(1)**  **Never** | | | | **(2)**  **Very Rare** | | | | **(3)**  **Occasionally** | | | | **(4)**  **Mostly** | | | | **(5)**  **Always** | |  |
| **D. Communication** |  | | | |  | | | |  | | | |  | | | |  | |  |
| Benefits from clear and effective communication methods and tools while communicating with teachers. (29) | ***(*** | | ***)*** | | ***(*** | | ***)*** | | ***(*** | | ***)*** | | ***(*** | | ***)*** | | ***( )*** | |  |
| Does works for the development of communication between staff. | ***(*** | | ***)*** | | ***(*** | | ***)*** | | ***(*** | | ***)*** | | ***(*** | | ***)*** | | ***( )*** | | **)** |
| Promptly notifies teachers of any change in the legislations and application. | ***(*** | | ***)*** | | ***(*** | | ***)*** | | ***(*** | | ***)*** | | ***(*** | | ***)*** | | ***( )*** | |  |
| Informs teachers regarding newly received directions and procedures. | ***(*** | | ***)*** | | ***(*** | | ***)*** | | ***(*** | | ***)*** | | ***(*** | | ***)*** | | ***( )*** | |  |
| Uses vertical and horizontal communication types in the institution. | ***(*** | | ***)*** | | ***(*** | | ***)*** | | ***(*** | | ***)*** | | ***(*** | | ***)*** | | ***( )*** | |  |
| Ensures that a warm and friendly atmosphere is created between staff relations within the institution. (34) | ***(*** | | ***)*** | | ***(*** | | ***)*** | | ***(*** | | ***)*** | | ***(*** | | ***)*** | | ***( )*** | |  |
| Follows the course of any activities organized in the institution and consult with teachers about any observed deficiencies and measures to be taken. | ***(*** | | ***)*** | | ***(*** | | ***)*** | | ***(*** | | ***)*** | | ***(*** | | ***)*** | | ***( )*** | |  |
| Allow teachers to express their ideas and opinions clearly within the institution. | ***(*** | | ***)*** | | ***(*** | | ***)*** | | ***(*** | | ***)*** | | ***(*** | | ***)*** | | ***( )*** | |  |
| Ensures that the wishes and complaints of staff in charge of teachers are easily conveyed to the institutions management. | ***(*** | | ***)*** | | ***(*** | | ***)*** | | ***(*** | | ***)*** | | ***(*** | | ***)*** | | ***( )*** | |  |
| Benefits from clear and effective communication methods and tools while communicating with teachers. | ***(*** | | ***)*** | | ***(*** | | ***)*** | | ***(*** | | ***)*** | | ***(*** | | ***)*** | | ***( )*** | |  |
| **Your Institution Manager;** | **(1)**  **Never** | | | | **(2)**  **Very Rare** | | | | **(3)**  **Occasionally** | | | | **(4)**  **Mostly** | | | | **(5)**  **Always** | |  |
| **E. Coordination** |  | | | |  | | | |  | | | |  | | | |  | |  |
| Ensures that all the institutions members participate and play an active and productive role in the education mechanism of the institution. (39) | ***(*** | ***)*** | | | ***(*** | ***)*** | | | ***(*** | ***)*** | | |  | ***( )*** | | |  | ***( )*** |  |
| Encourage cooperation between teachers, other employees, students, parents, school council and top-level managers to assist in the institution reaching its goals. | ***(*** | ***)*** | | | ***(*** | ***)*** | | | ***(*** | ***)*** | | |  | ***( )*** | | |  | ***( )*** |  |
| Schedules educational activities for teachers (monthly-daily plans, parent meetings, family education and celebrating special days) and ensure that all the activities are carried out collaboratively. (41) | ***(*** | ***)*** | | | ***(*** | ***)*** | | | ***(*** | ***)*** | | |  | ***( )*** | | |  | ***( )*** |  |
| Prioritize in-service education and ensure that all the staff members are trained well in this field. (42) | ***(*** | ***)*** | | | ***(*** | ***)*** | | | ***(*** | ***)*** | | |  | ***( )*** | | |  | ***( )*** |  |
| Plays a conciliatory role between teachers, top-level managers (ex. director of national education) students, parents and institution staff. | ***(*** | ***)*** | | | ***(*** | ***)*** | | | ***(*** | ***)*** | | |  | ***( )*** | | |  | ***( )*** |  |
| Informs institution employees regarding each other’s work. | ***(*** | ***)*** | | | ***(*** | ***)*** | | | ***(*** | ***)*** | | |  | ***( )*** | | |  | ***( )*** |  |
| Attach importance to studies that increase cooperation in the institution. | ***(*** | ***)*** | | | ***(*** | ***)*** | | |  | ***( )*** | | |  | ***( )*** | | |  | ***( )*** |  |
| **Your Institution Manager;** | **(1)**  **Never** | | | | **(2)**  **Very Rare** | | | | **(3)**  **Occasionally** | | | | **(4)**  **Mostly** | | | | **(5)**  **Always** | |  |
| **F. Influence** |  | | | |  | | | |  | | | |  | | | |  | |  |
| Uses reward as a motivation source to boost/increase teachers’ success. (46) | ***(*** | ***)*** | | | ***(*** | ***)*** | | |  | ***( )*** | | |  | ***( )*** | | | ***(*** | ***( )*** |  |
| Uses menace and punishment to motivate teachers. | ***(*** | ***)*** | | | ***(*** | ***)*** | | |  | ***( )*** | | |  | ***( )*** | | | ***(*** | ***( )*** |  |
| Uses both material and moral rewarding to motivate teachers. (48) | ***(*** | ***)*** | | | ***(*** | ***)*** | | |  | ***( )*** | | |  | ***( )*** | | | ***(*** | ***( )*** |  |
| Assists teachers and other employees about embracing their work and fully committing themselves to the institution. | ***( )*** | | | | ***( )*** | | | | ***( )*** | | | | ***( )*** | | | | ***( )*** | |  |
| Finds immediate and effective solutions to the problems experienced within the institution. | ***( )*** | | | | ***( )*** | | | | ***( )*** | | | | ***( )*** | | | | ***( )*** | |  |
| Motivates teachers for using various education methods and techniques. | ***( )*** | | | | ***( )*** | | | | ***( )*** | | | | ***( )*** | | | | ***( )*** | |  |
| Refer to social and psychological influencing methods rather than using my authority. | ***( )*** | | | | ***( )*** | | | | ***( )*** | | | | ***( )*** | | | | ***( )*** | |  |

| **Your Institution Manager;** | **(1)**  **Never** | | **(2)**  **Very Rare** | | **(3)**  **Occasionally** | | **(4)**  **Mostly** | | **(5)**  **Always** | |
| --- | --- | --- | --- | --- | --- | --- | --- | --- | --- | --- |
| **G. Evaluation** |  | |  | |  | |  | |  | |
| Monitors and evaluates teacher’s lectures and work in terms of their efficiency at various times of the academic year. (53) | ***(*** | ***)*** | ***(*** | ***)*** | ***(*** | ***)*** | ***(*** | ***)*** | ***(*** | ***)*** |
| Carries out evaluation with teachers and prepare regulations to keep the institution up-to-date modern and more effective. | ***(*** | ***)*** | ***(*** | ***)*** | ***(*** | ***)*** | ***(*** | ***)*** | ***(*** | ***)*** |
| Implements the results of inspections and evaluations together with teachers. | ***(*** | ***)*** | ***(*** | ***)*** | ***(*** | ***)*** | ***(*** | ***)*** | ***(*** | ***)*** |
| Oversees the time keeping of teacher’s scheduled activities, including the consistency and harmony of these activities. | ***(*** | ***)*** | ***(*** | ***)*** | ***(*** | ***)*** | ***(*** | ***)*** | ***(*** | ***)*** |
| Evaluates teachers objectively and equally. | ***(*** | ***)*** | ***(*** | ***)*** | ***(*** | ***)*** | ***(*** | ***)*** | ***(*** | ***)*** |
| Displays constructive and developing behavior when delivering results of an audit and its evaluation. | ***(*** | ***)*** | ***(*** | ***)*** | ***(*** | ***)*** | ***(*** | ***)*** | ***(*** | ***)*** |
| Sees the institution as a whole and assess and evaluate the effectiveness and efficiency level of the school. | ***(*** | ***)*** | ***(*** | ***)*** | ***(*** | ***)*** | ***(*** | ***)*** | ***(*** | ***)*** |
| Makes objective and motivating evaluations when evaluating the personnel of an institution. | ***(*** | ***)*** | ***(*** | ***)*** | ***(*** | ***)*** | ***(*** | ***)*** | ***(*** | ***)*** |
| Supervises teacher’s motivation to work efficiently. (61) | ***(*** | ***)*** | ***(*** | ***)*** | ***(*** | ***)*** | ***(*** | ***)*** | ***(*** | ***)*** |

***Interview Date: ………………………………….***

***Interview Place: ……………………………......***

***Your Institutions’ Name: …………………………***
